# Editorial: Refractory and relapsed hemophagocytic lymphohistiocytosis in pediatric population: targeted therapy

**DOI:** 10.3389/fonc.2025.1691878

**Published:** 2025-09-12

**Authors:** Madalina Bota, Şefik Akyol

**Affiliations:** ^1^ University of Medicine and Pharmacy Iuliu Hatieganu, Cluj-Napoca, Romania; ^2^ Antalya Research and Training Hospital, Health Sciences University, Antalya, Türkiye

**Keywords:** hemophagocytic lymphohistocytosis, cancer, children, target therapy, rheumatic disease

Hemophagocytic lymphohistiocytosis is a complex and severe disease. Although rare, its highly lethal hyperinflammatory syndrome driven by uncontrolled immune activation, makes early consideration of the diagnosis critical in order to accelerate the diagnostic work-up and initiate timely treatment. Several causes are known to determine the occurrence of this disease, starting from genetic causes and ending with several infections, malignancy or rheumatological diseases. Due to its variability, treating the disease might be difficult. First-line treatment for secondary HLH is based on immunosuppression with dexamethasone and cyclosporine, associated with etoposide. Primary HLH or severe secondary HLH will need consolidation with HSCT. In most cases remission is achieved, but there still are severe forms that are either refractory from the beginning or relapse after a first remission and need an individualized approach.

In recent years, there has been a paradigm shift in the management of HLH. Alongside conventional immunosuppressive regimens, personalized treatment approaches targeting the underlying etiology and molecular pathogenesis have gained increasing attention. Targeted therapies have ushered in a new era in HLH management. Agents such as JAK–STAT pathway inhibitors, interferon-gamma blockade, and cytokine-modulating biologics have shown promising results in refractory or relapsed cases. These strategies not only suppress the hyperinflammatory state but also aim to correct the underlying immune dysregulation, offering the potential for more durable remissions.

This Research Topic aimed to outline: (1) the variability in epidemiological aspects, (2) the difficulty of individualized therapy for patients, (3) the high incidence of deaths in refractory and relapsed cases, and not the last (4) the need for more extensive studies for targeted therapies in HLH in the pediatric population.

Wang et al. describe a large cohort of 52 patients that have been treated over a period of 18 years in a single center. Several etiologies were confirmed, with infection associated HLH being the most common, seconded by HLH-associated to rheumatic diseases. There were also a few cases induced by malignancy, one patient with familial HLH with UNC13D mutation, one case with Chediak-Higashi syndrome and two patients with X-linked lymphoproliferative disease type 1 and type 2. First-line treatment was used according to the HLH-2004 protocol, but there was still the need for targeted therapies in some of the cases. Rituximab was used for EBV positive cases, tocilizumab and adalimumab were used for JIA-induced HLH, ruxolitinib was used for the patient with XLPD type 2. Despite aggressive therapy, mortality rate remained high, up to 30.8%, suggesting that new studies need to be performed in order to raise the chance for complete and long-term remission for patients with refractory and relapsed HLH.

The case report presented by Zou et al. shows that HLH can be met also in solid tumors, such as Ewing sarcoma, even though malignancy HLH is usually associated with leukemias and lymphomas. The criteria for the diagnosis of HLH might overlap on the diagnosis of malignancy making it harder for the physician to confirm an early diagnosis of associated HLH. The inability to control the underlying cause leads to a refractory form of HLH, and intensive immunosuppression might lead to severe infections, as was the case of this patient. The balance of treating both the cause and the HLH as a complication is very hard to keep, which is why more reports are needed in order to set a more standardized approach in these cases.

Zheng et al. report a rare case of the Hodgkin lymphoma variant of Richter syndrome (HL-type RS) presenting with hemophagocytic syndrome. This represents the first documented instance of clonally unrelated HL-type RS, confirmed through detailed immunoglobulin gene rearrangement and mutation analyses. The patient, with prior small lymphocytic lymphoma and recent Zanubrutinib therapy, responded favorably to a modified R-ABVD regimen combined with Zanubrutinib. This case underscores the importance of molecular clonality assessment in HL-type RS and expands our understanding of its clinical spectrum and therapeutic options.

Pawińska-Wąsikowska et al. describe the first reported case of concurrent acute lymphoblastic leukemia (ALL), disseminated juvenile xanthogranuloma (JXG), and hemophagocytic lymphohistiocytosis (HLH). Molecular analysis confirmed a clonal relationship between leukemic blasts and histiocytes in skin and bone marrow, suggesting a common cellular origin. Despite multimodal therapy including corticosteroids, vinblastine, etoposide, cyclosporine, tocilizumab, and eventual hematopoietic stem cell transplantation, bone marrow recovery remained limited, while skin lesions improved markedly after empirical antitubercular treatment. This unique case emphasizes the heterogeneity of histiocytic disorders, potential infectious triggers of HLH, and the need for integrated diagnostic and therapeutic approaches.

The evolving landscape of HLH diagnosis and management underscores the critical need for early recognition, precision medicine, and collaborative care. Advances in molecular diagnostics and targeted therapeutics are transforming HLH from a uniformly fatal condition to a potentially controllable disease in selected patients. Future research should focus on refining risk stratification tools, identifying predictive biomarkers, and integrating novel agents into standardized treatment algorithms. Given the complexity and heterogeneity of HLH, optimal patient outcomes will require a multidisciplinary approach involving hematologists, immunologists, infectious disease specialists, and critical care teams.

